# GP characteristics and video use in out-of-hours primary care: a register-based study

**DOI:** 10.3399/BJGPO.2024.0269

**Published:** 2025-12-19

**Authors:** Ida Bergholdt Jul Christiansen, Mette Amalie Nebsbjerg, Claus Vestergaard, Katrine Bjørnshave Bomholt, Morten Bondo Christensen, Linda Huibers

**Affiliations:** 1 Research Unit for General Practice, Aarhus, Denmark; 2 Department of Internal Medicin, Randers Regional Hospital, Randers, Denmark; 3 Department of Public Health, Aarhus University, Aarhus, Denmark

**Keywords:** after-hours care, remote consultation, general practice

## Abstract

**Background:**

GPs can use video when performing telephone triage in out-of-hours primary care (OOH-PC) in Denmark. Video use varies considerably among GPs; this variation could be related to GP characteristics.

**Aim:**

To investigate associations between GP characteristics and video use in OOH-PC telephone triage.

**Design & setting:**

A register-based study using data from the OOH-PC registration system from 1 January 2021 to 31 December 2021.

**Method:**

Binomial regression analysis was used to measure the associations between video contacts and triage GP characteristics, thereby calculating risk ratios (RRs) and 95% confidence intervals (CI).

**Results:**

Video was used in 10.8% of telephone triage contacts to OOH-PC. Video use was significantly associated with GPs having more shifts in OOH-PC (RR: 1.36–1.93, reference: low number of shifts) and GPs being younger (RR: 0.84–0.67, reference: age<40 years). Central Denmark Region and Region of Southern Denmark had significant higher video-user rates (RR: 1.23–1.46) than North Denmark Region, whereas Region Zealand had significant lower rates (RR = 0.57, 95% confidence interval [CI] = 0.38 to 0.87). The association between video use and GP sex was modified by number of shifts in OOH-PC. Video use was positively correlated with male sex among GPs with low, medium, and high number of shifts (RR = 1.18, 95% CI = 1.07 to 1.29) and negatively correlated with male sex among GPs with very high number of shifts (RR = 0.75, 95% CI = 0.58 to 0.98).

**Conclusion:**

Video use was associated with the number of shifts in OOH-PC, GP sex and age, and geographical region.

## How this fits in

Video is increasingly used in telephone triage in out-of-hours primary care (OOH-PC). As video use is known to depend on context, GP characteristics could predict whether a triage GP will choose video. Video use was associated with GP age and sex, geographical region, and number of shifts. Future research should further explore GP characteristics such as personality traits.

## Introduction

Video use has become widespread as part of a political drive for reorganisation and digitalisation of healthcare services,^1–6^ and pushed even further during the COVID-19 pandemic.^3,6–8^ Video enables healthcare staff to perform remote visual assessment of patients, supporting them to refer patients to the right care at the right time.^9–16^ Primary care is facing challenges, such as increased demand and workforce shortages,^5,17–21^ and video could possibly contribute to more sustainable primary health care.^9,14–16^

Video use in health care is complex^22^ and highly context-dependent,^6,23–28^ as described in the Planning and Evaluating Remote Consultation Services (PERCS) framework.^24^ PERCS describes eight domains (that is, reasons for consulting, patient, clinical relationship, home and family, technologies, staff, healthcare organisation, and wider system) that interact and evolve over time.

Most research within the healthcare staff domain focuses on staff views.^6,12,22,23,25,29^ Gren *et al* found that triage nurses at a Danish out-of-hours primary care (OOH-PC) service felt more reassured about their triage decisions, and found it easier to reassure parents after using video in consultations concerning children.^12^ Studies reported that GPs were positive towards video use in OOH-PC.^10,13,25^ The GPs found video particularly suitable for urgent care, as it provides additional anamnestic information and helps prioritise patients who need further care. In daytime general practice, GPs have found video beneficial owing to fast access to health care and flexibility in care delivery.^30–33^ However, GPs have described barriers to video use, such as technical issues, concerns regarding practice organisation, and insecurities about which patients benefit from video use.^22,29^ Results on video use from daytime general practice are not directly transferable to the OOH-PC. In OOH-PC, video is primarily used as a triage tool that provides visual information to assist the triage professional and the focus is on acute single-health problems.^19,28,34–36^ In contrast, during daytime, video is used to substitute a face-to-face consultation. Video in OOH-PC is unidirectional, thus mainly adding information for the triage decision, whereas bidirectional use of video in daytime can support the relational aspects of care.^28,37^

Limited knowledge exists on healthcare staff characteristics related to video use. One Australian study found that GPs' video use in daytime was higher among GPs with more nurses in their practice and with prior video experience. Whereas lower video use was found among older GPs, GPs working long hours, and GPs in rural and larger daytime practices.^27^ However, generalisability of results to other countries are limited.

### Aim

We aimed to investigate associations between video use and GP characteristics (for example, age, sex, seniority) in a Danish OOH-PC setting. We hypothesised that GP characteristics are associated with video use.

## Method

### Design and population

We conducted a register-based study with data from the OOH-PC registration systems and the Danish Authorisation Register. We included all telephone triage contacts to OOH-PC in four out of five Danish regions from 1 January 2021 to 31 December 2021. The Capital Region of Denmark operates a different OOH-PC model not comparable with the models in the other four Danish regions and is not included.

### Setting

Primary care is free (tax-funded) in Denmark, with access for all residents. GPs act as gatekeepers for secondary care. OOH-PC services are open on weekdays between 4.00 pm and 8.00 am and during weekends and holidays. GPs are obligated to cover shifts in the regional OOH-PC service. Some GPs primarily work at the OOH-PC service. Patients must always first contact the OOH-PC by phone. GPs perform telephone triage and decide to give telephone advice, refer to a face-to-face consultation with a GP (that is, clinic consultation or home visit), or refer directly to the hospital.

In March 2020, video was introduced in the OOH-PC in three Danish regions (the North Denmark region, the Central Denmark Region, and the Region of Southern Denmark) owing to the COVID-19 pandemic and a year later in Region Zealand.

During triage, GPs have the option to use video, which is a video contact. If the GP finds a health problem suitable for video use, a video link is sent to the caller in a text message. When activated the GP can see the patient, but the patient cannot see the GP. If the GP does not use video, the contact is a telephone contact. GPs are remunerated by fee-for-service; as video contacts take more time, the fee is higher for video than for telephone contacts.

### Outcome measures

The following two outcome measures were defined: proportion of video contacts per telephone triage contacts (to measure video-user rate among GPs in OOH-PC) and risk ratios (RRs) (to measure the association between video use and GP characteristics in OOH-PC).

### Data collection and data management

The regional OOH-PC registration system provided information about type of contact (that is, telephone or video contact) and GP age and sex. The Danish Authorisation Register provided information on specialisation (that is, type and date) and authorisation date. Data were linked through the GP’s authorisation number, which is a unique number granted to all healthcare professionals by the authorities.

We calculated the number of telephone triage shifts within the past 180 days to achieve a measurement of the individual GP’s familiarity with telephone triage in OOH-PC. This variable (number of OOH shifts) was categorised into the following four groups: 1–2 shifts (low); 3–12 shifts (medium); 13–20 shifts (high); and 31–148 shifts (very high). GP seniority was defined as the number of years since GP specialisation and categorised into the following four groups: GP trainee; 0–5 years; 6–10 years; and>10 years. GP age was categorised into the following five groups:<40 years; 40–49 years; 50–59 years; 60–69 years; and 70–75 years.

**Figure 1.**
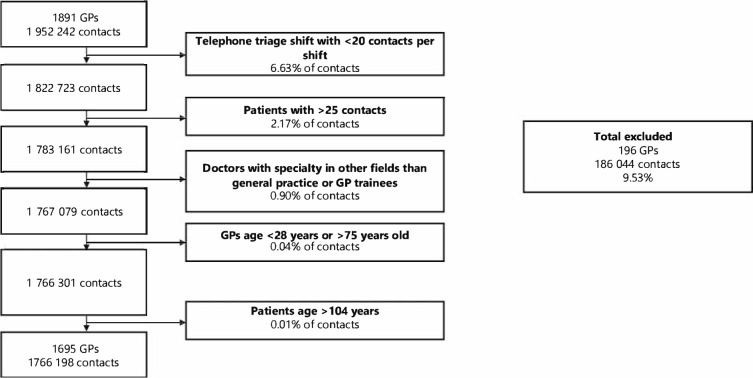
Flowchart: data cleaning

Several types of contacts were excluded (Figure 1). We aimed to include only GPs in telephone triage shifts with access to video, thereby excluding GPs in other shifts who supported telephone triage during peak times. We defined a telephone shift as >20 consecutive telephone triage contacts (6.6% of all telephone triage contacts excluded). Patients with >25 contacts to OOH-PC during the study period were considered outliers. These frequent callers are often citizens with mental health issues who contact OOH-PC multiple times a day. These contacts were excluded (2.2%) to focus on the majority of users. We identified eight physicians who were neither GPs nor GP trainees according to the Danish Authorisation Register (0.9% excluded). As Danish GPs must reapply for authorisation after the age of 75 years, triage GPs aged >75 years (*n* = 11) were considered a specific and small group and we excluded them to focus on the majority of GPs (<0.1% excluded). Patients aged >104 years were considered registration errors (<0.1% excluded). Thus, the data cleaning process resulted in a total number of 1 766 198 contacts (9.5% excluded), which corresponded to a total number of 1695 GPs (9.8% excluded).

### Analysis

We conducted descriptive analyses stratified for type of contact. Binomial regression analysis was used to measure the associations between video contacts and triage GP characteristics, thereby calculating RRs and 95% confidence intervals (95% CIs). We allowed for repeated measurements by applying the vce option in Stata to account for clustering at GP level. Furthermore, we performed crude and adjusted analyses (adjusting for sex, age, and region). Results were presented in forest plots. To investigate effect modification by the group of GPs who covered a very high number of shifts, we performed stratified analyses, both limited to this group and without this group. The distribution of patients is random. Therefore, we did not take patient characteristics into account as possible confounders. Stata (version 16) was used for all analyses.

## Results

### Study population

In total, 1 766 198 triage contacts were conducted within the study period (Table 1). The video-user rate was 10.8%, varying between 5% and 15% in three out of four regions during the entire study period. Owing to late introduction of video, Region Zealand did not reach a 5% video-user rate until July 2021 (data not shown).

**Table 1.** Distribution of GP characteristics for triage contacts, stratified for type of contact

	**Triage contacts, *n* (%)**		**Total contacts**
	**Telephone contacts**	**Video contacts**	
**GP characteristics**	1 575 246 (89.2)	190 952 (10.8)	1 766 198
**GP sex**			
Female	541 726 (90.0)	60 189 (10.0)	601 915
Male	1 033 520 (88.8)	130 763 (11.2)	1 164 283
**GP age, years**			
<40	153 011 (86.5)	23 970 (13.5)	176 981
40–49	544 862 (88.6)	70 014 (11.4)	614 876
50–59	432 412 (89.9)	48 846 (10.1)	481 258
60–69	371 073 (90.9)	37 135 (9.1)	408 208
70–75	73 888 (87.1)	10 987 (12.9)	84 875
**Region of Denmark**			
North Denmark Region	202 074 (90.6)	21 007 (9.4)	223 081
Central Denmark Region	523 344 (88.4)	68 432 (11.6)	591 776
Region of Southern Denmark	518 998 (86.3)	82 665 (13.7)	601 663
Region Zealand	330 830 (94.6)	18 848 (5.4)	349 678
**Number of shifts^a^**			
Low	99 013 (93.1)	7290 (6.9)	106 303
Medium	554 201 (90.7)	57 106 (9.3)	611 307
High	476 829 (89.1)	58 518 (10.9)	535 347
Very high	445 203 (86.7)	68 038 (13.3)	513 241
**Seniority, years^b^**			
GP trainee	124 280 (86.0)	20 267 (14.0)	144 547
0–5	274 375 (88.4)	36 047 (11.6)	310 422
6–10	250 394 (88.9)	31 224 (11.1)	281 618
>10	926 197 (90.0)	103 414 (10.0)	1 029 611

^a^ *Number of shifts: number of telephone triage shifts in OOH-PC within the 180 days before each contact: low = 1–2, medium = 3–12, high = 13–30, very high = 31–148*. ^b^ *Seniority: number of years since achieved GP specialisation*. OOH-PC = out-of-hours primary care

### Associations between GP characteristics and video use

**Figure 2.**
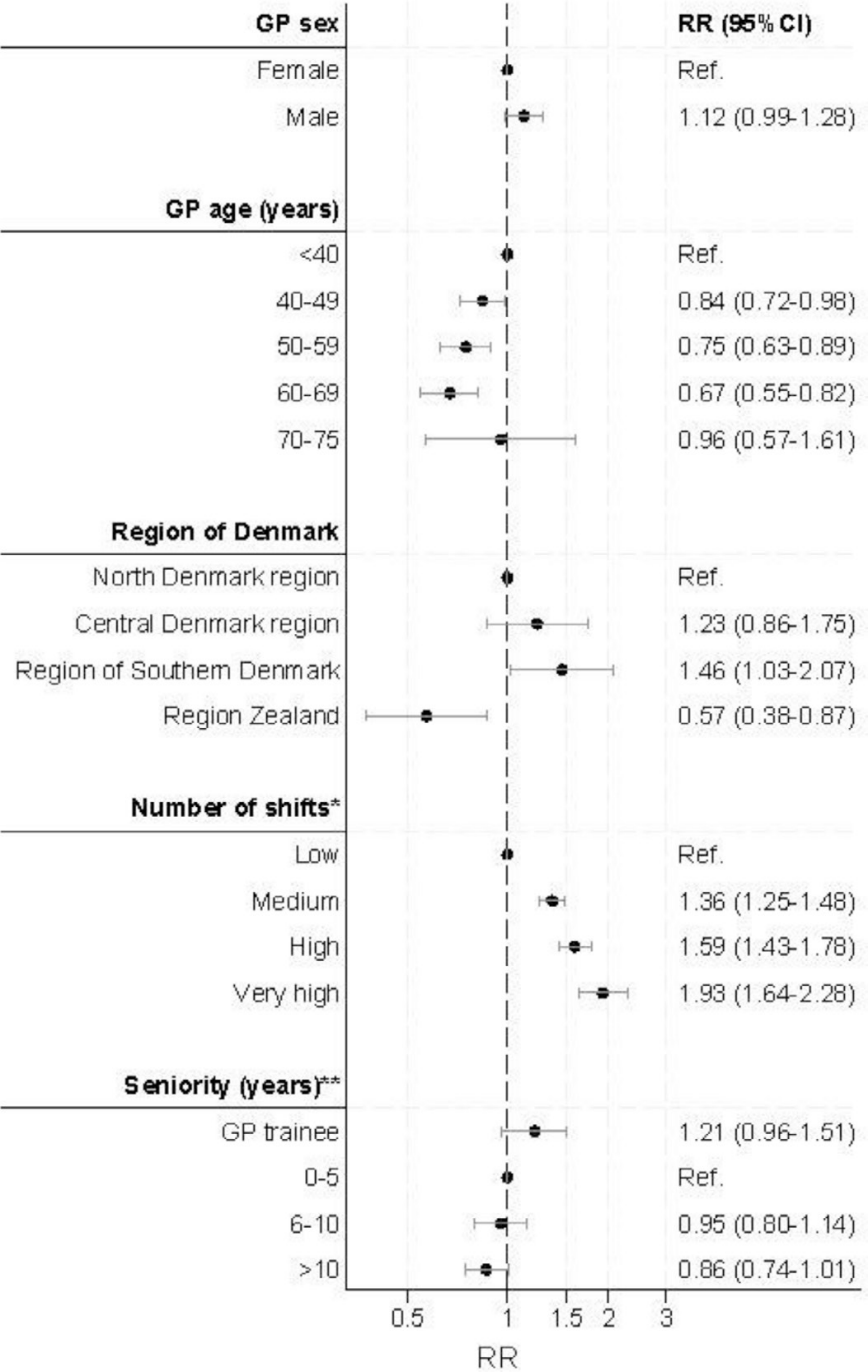
Crude association between GP characteristics and video use in OOH-PC (RR, 95% CI). ^a^Number of shifts: number of telephone triage shifts in OOH-PC during the 180 days before each contact: low = 1–2, medium = 3–12, high = 13–30, very high = 31–148 telephone shifts. ^b^Seniority: number of years since achieved specialisation. CI = confidence interval. RR = risk ratio. OOH-PC = out-of-hours primary care

The RRs of video contacts varied with GP characteristics (Figure 2). We found a dose-response relationship between GP age and video use. GPs aged <40 years had significantly higher video use compared with GPs aged 40–69 years (RR = 0.84–0.67, reference: age <40 years). GPs aged 70–75 years had similar video-user rates as GPs aged <40 years (RR = 0.96, 95% confidence interval [CI] = 0.57 to 1.61). GPs from the Central Denmark Region (RR = 1.23, 95% CI = 0.86 to 1.75) and the Region of Southern Denmark (RR = 1.46, 95% CI = 1.03 to 2.07) had significantly higher video use than GPs from the North Denmark Region, whereas GPs from Region Zealand (RR = 0.57, 95% CI = 0.38 to 0.87) had significantly lower video use than GPs from the North Denmark Region.

Our data showed a dose-response relationship between number of shifts and video use, as we found higher video use among GPs with more shifts (RR = 1.36–1.93, ref: low number of shifts). However, we did not find a significant association between GP sex and video use in our crude analysis (RR = 1.12, 95% CI = 0.98 to 1.28). Adjusted estimates (that is, adjusted for number of shifts, GP age, seniority, and region) differed only marginally (Supplementary Figure 1).

**Figure 3.**
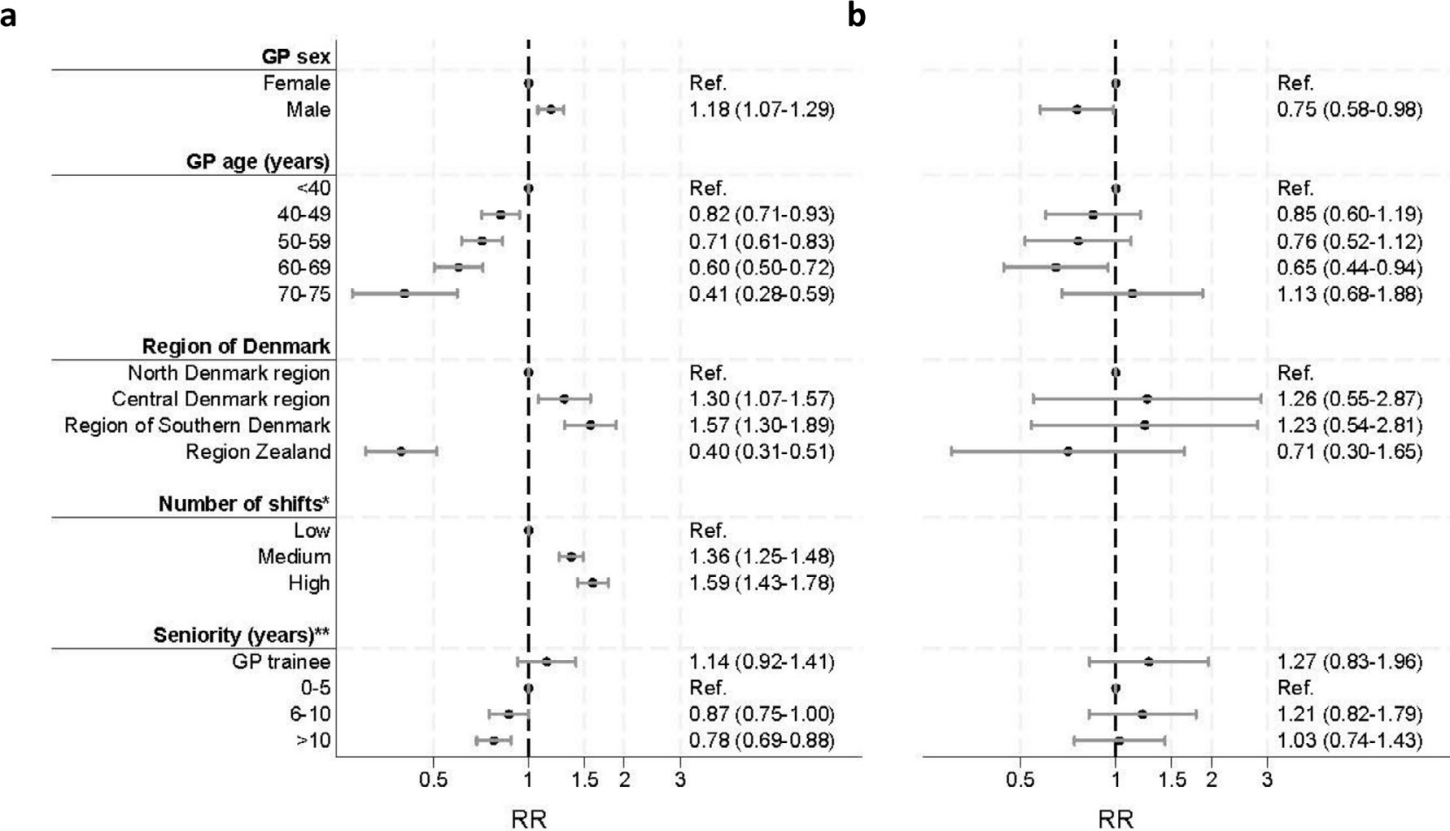
Crude association between GP characteristics and video use in OOH-PC (RR, 95% CI), stratified for number of shifts a. GPs with low, medium, and high number of shifts; b. GPs with very high number of shifts. *Number of shifts: number of telephone triage shifts in OOH-PC during the 180 days before each contact: low = 1–2, medium = 3–12, high = 13–30, very high = 31–148 telephone shifts. **Seniority: number of years since achieved specialisation. CI = confidence interval. RR = risk ratio. OOH-PC = out-of-hours primary care

To investigate the estimates for the GPs aged 70–75 years, we checked for effect modification, performing stratified analyses by number of shifts (Figure 3). When only including contacts of GPs who had fewer than ‘very high number of shifts’ (Figure 3a), the estimate for the GPs aged 70–75 years (RR = 0.41, 95% CI = 0.28 to 0.59) aligned with the dose-response relation seen for the other age groups. This stratification by number of shifts seemed to change the direction of the relation between video use and GP sex: for GPs with very high number of shifts, male GPs used video less than female GPs (RR = 0.75, 95% CI = 0.58 to 0.98), whereas male GPs used video more in the group with fewer shifts (RR = 1.18, 95% CI = 1.07 to 1.29). Thus, the number of shifts modified the effect of age and sex and was a strong modifier of video use among GPs. GPs with very high number of shifts had a higher use of video in OOH-PC, which modified the general effect of less video use among older GPs.

## Discussion

### Summary

Video was used in 10.8% of triage contacts to the OOH-PC. We found significant associations between video use and GP characteristics. Large number of telephone triage shifts in OOH-PC, young age (<40 years), and working in the Central Denmark Region or the Region of Southern Denmark were positively associated with video use.

### Strengths and limitations

The data were based on coding for remuneration purposes, including registration of video use, which supports completeness of data owing to the economic incentive.^38^

The study had some limitations. First, our dataset included a specific subgroup of GPs aged 70–75 years. However, we were able to correct for this in our stratified analysis. Second, the COVID-19 pandemic was still ongoing in our study period, which might have influenced our results. The pandemic worked as a strong incentive towards video use, but it is unlikely that it acted as a confounding factor in the association between GP characteristics and video use. As video use remained stable after the pandemic, we assume that possible associations between GP characteristics and video use have not changed considerably. Third, as only register-based data were available, we lacked relevant information about additional GP characteristics that could have impacted video use, such as GP personality traits (for example, risk behaviour and/or digital literacy). In certain groups of GPs (for example, younger age), we hypothesise that differences in risk behaviour and digital literacy may influence the frequency of video use, making these factors potential confounders. Fourth, we studied one-way video with GP-led triage, whereas telephone triage in OOH services is performed by nurses in most other countries.^39^ Finally, compared with other international studies, we found a higher percentage of video use, which may be attributed to the differences in the organisation of OOH-PC. The remuneration for a video contact was higher than that for a telephone contact as video is more time-consuming. Yet, this could have added an economic incentive to use video, which may have contributed to the higher percentage of video use observed compared with other international studies.^27^

### Comparison with existing literature

To the best of our knowledge, this is the first study to investigate the association between video use and GP characteristics in an OOH-PC setting. Two studies, from a daytime general practice setting, found that video use was highly associated with GP characteristics.^27,40^ Rodriguez *et al* found that clinician characteristics accounted for about one-quarter of the variance in video use.^40^ Scott *et al* found that GPs aged >55 years used video less often than their younger GP colleagues,^27^ which is largely in line with our results. While overall analysis suggested GPs aged 70–75 years had rather high levels of video use, stratified analysis showed that this was only true for a particular subset of this group, namely those with ‘very high number of shifts’. As most GPs are retired at this age, this subgroup of older GPs probably differed from the overall population of GPs, for example, by being more inclined to work with new technology or having specific personality traits. The findings of these studies are difficult to compare with the Danish context, as the organisation of healthcare systems differs considerably.

A large study from the UK, which was based on interviews with 55 GPs, found that GP attitudes towards using video in OOH were influenced by their experience with video, including previous use of video during the daytime.^25^ Two qualitative studies reported that GPs found the use of video more meaningful in the OOH-PC setting compared with the daytime setting.^13,25^ One could hypothesise that GPs with many shifts in OOH-PC are generally more familiar with video and thus use it more frequently. Other possible explanations may exist. GPs who have a very high number of shifts may have higher financial incentive as the OOH work is their main income.

We found differences in video use between the Danish regions. We suspect that these differences are related to organisational differences. Although Denmark is largely homogeneous, between-region and in-region differences exist in the demographic characteristics of the population and in the organisation of GP clinics. Some of these differences may partly explain our findings, such as lower number of daytime GPs, larger GP shortage, and larger number of solo practices in the North Denmark Region and Region Zealand compared with the Central Denmark Region and the Southern Denmark Region. Moreover, Scott *et al* found an association between practice organisation and video use, as less video use was seen for GPs in solo practices.^27^

Level of seniority and video use in OOH-PC seemed to have a dose-response relation, with reduced use with higher seniority. In contrast to our results, an Australian study conducted in a daytime setting found that GPs who practised longer were less likely to express negative views towards video use.^40^ Whereas Scott *et al* found no association between sex and video use,^27^ our results suggested a positive association between video use and male sex. However, this association had a reverse direction for GPs with very high number of shifts; here female GPs used video more often than male GPs.

### Implications for research and practice

Our findings add insight into the understanding of video use in OOH-PC telephone triage. Previous studies have described the view of staff members on video use,^6,10,12,13,22,23,25^ whereas our study adds knowledge about GP characteristics and the association with using video.

This study suggests that variation in video use is at least partly driven by individual GPs’ characteristics and not just by evidence-based criteria. GPs who take more shifts in OOH-PC may have developed relevant ways of using one-way video as a triage tool. Exploring their experiences could provide valuable insights for better integration of video use.

Future research should investigate personal characteristics related to video use, for example, GP personality traits such as risk behaviour and digital literacy, using qualitative or questionnaire-based methods.

Moreover, investigating video use among other groups of triage professionals is highly relevant.

In conclusion, the study found that GP characteristics are associated with video use. Video use was associated with larger number of shifts at the OOH-PC, being of young age (<40 years), and male sex. Future research should include GP personality traits such as risk adversity.

## Data

The dataset relied on in this article is available from the corresponding author on reasonable request.
